# Self-Modulated Ghost Imaging in Dynamic Scattering Media

**DOI:** 10.3390/s23219002

**Published:** 2023-11-06

**Authors:** Ying Yu, Mingxuan Hou, Changlun Hou, Zhen Shi, Jufeng Zhao, Guangmang Cui

**Affiliations:** 1 Institute of Carbon Neutrality and New Energy, School of Electronics and Information, Hangzhou Dianzi University, Hangzhou 310018, China; yy18845032278@163.com (Y.Y.); hmxgzyx@163.com (M.H.); shizhen@hdu.edu.cn (Z.S.); dabaozjf@hdu.edu.cn (J.Z.); cuigm@hdu.edu.cn (G.C.); 2 Zhejiang Province Key Laboratory of Equipment Electronics, Hangzhou 310018, China

**Keywords:** ghost imaging, dynamic scattering imaging, self-modulated ghost imaging

## Abstract

In this paper, self-modulated ghost imaging (SMGI) in a surrounded scattering medium is proposed. Different from traditional ghost imaging, SMGI can take advantage of the dynamic scattering medium that originally affects the imaging quality and generate pseudo-thermal light through the dynamic scattering of free particles’ Brownian motion in the scattering environment for imaging. Theoretical analysis and simulation were used to establish the relationship between imaging quality and particle concentration. An experimental setup was also built to verify the feasibility of the SMGI. Compared with the reconstructed image quality and evaluation indexes of traditional ghost imaging, SMGI has better image quality, which demonstrates a promising future in dynamic high-scattering media such as dense fog and turbid water.

## 1. Introduction

Scattering media are widely present in nature, which hinders the direct analysis of scene information from traditional optical systems. Image reconstruction through dynamic and complex scattering media has always been a hot topic [1,2,3,4,5,6,7]. A large body of research has historically focused on compensating or straight-out eliminating scattering effects [8,9], terahertz imaging [10,11], etc. Ghost imaging (GI) is based on the Hanbury Brown–Twiss (HBT) effect and reconstructs images by calculating the correlation between the object and reference beams [1,12]. Ghost imaging has potential applications in many fields [13], such as optical encryption [14,15,16], radar imaging [17,18,19], and three-dimensional imaging [20,21,22]. GI has also been demonstrated to have the advantage of imaging in scattering media, as it can effectively reduce the interference of the scattering medium and obtain a clearer image in this medium [23,24]. At present, most of the research on ghost imaging in scattering media is focused on the influence of different scattering media on ghost imaging [25], the effect of scattering media in different paths on ghost imaging [26], and compensation reconstruction algorithms [27].

Speckle pattern and generation mode is one of the key factors affecting the quality of GI. Traditional GI uses a laser beam on a rotating ground glass to produce pseudo-thermal light [28]. Computational ghost imaging (CGI) requires devices such as SLMs or DMDs to generate random speckles with known rules [29]. However, they are often unable to withstand high light power. The refresh rate of the spatial light modulation device should be higher than the sampling rate of the bucket detector signal, or it will degrade the reconstructed image quality [30]. In some applications, such as GI at a remote distance, speckle patterns still need to be realized using rotating ground glass.

In this paper, we propose self-modulated ghost imaging (SMGI) in a scattering medium. Unlike the way of generating pseudo-thermal light in traditional ghost imaging, in SMGI, the pseudo-thermal light is produced through the dynamic scattering of free particles’ Brownian motion in the scattering environment. Simulation and experiment have verified that SMGI can be realized in a certain particle concentration environment. Compared with the image quality and evaluation indexes of traditional ghost imaging, SMGI improves the noise interference caused by the rotating ground glass and shows better performance.

## 2. Methods

### 2.1. GI Principle

**Figure 1:**
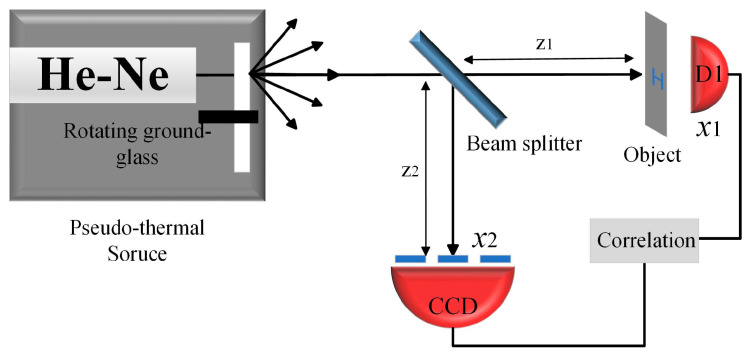
Ghost imaging experimental schematic diagram.

Let us briefly review the procedure of ghost imaging first. A typical schematic of classic pseudo-thermal light source ghost imaging is shown in Figure 1 [31,32]. A collimated laser beam is divided into two identical light beams through a beam splitter (BS). In the object arm, the light illuminates the object, and the transmitted light is collected by the bucket detector (D1), which is an energy detector without spatial resolution. A CCD camera records the light field in the reference arm. The distances of the beam splitter to the object surface and the beam splitter to the array detector are the same (z1 = z2). The light energy collected by the bucket detector D1 is

(1) $$S=\int I_{obj}(x_{1})T(x_{1})dx_{1}$$

where $I_{obj}(x_{1})$ is the intensity distribution of the illuminating light in the object arm and $T(x_{1})$ is the transmission function of the object. The object image is obtained by computing the second-order correlation, which can be expressed as:

(2) $$O\left(x\right)\propto \langle I_{ref}\left(x_{2}\right)S\rangle -\langle I_{ref}\left(x_{2}\right)\rangle \langle S\rangle =\int \langle I_{obj}\left(x_{1}\right)I_{ref}\left(x_{2}\right)\rangle [g^{\left(2\right)}\left(x_{1},x_{2}\right)-1]T(x_{1})d(x_{1})$$

where $\langle \cdot \rangle$ means multi-frame averaging, $I_{ref}(x_{2})$ is the intensity distribution of the speckle field in the reference path recorded using the CCD, and $g^{\left(2\right)}\left(x_{1},x_{2}\right)$ denotes the degree of second-order coherence defined as

(3) $$g^{\left(2\right)}\left(x_{1},x_{2}\right)=\frac{\langle I_{obj}\left(x_{1}\right)I_{ref}\left(x_{2}\right)\rangle }{\langle I_{obj}\left(x_{1}\right)I_{ref}\left(x_{2}\right)\rangle }$$

### 2.2. Self-Modulated GI

**Figure 2:**
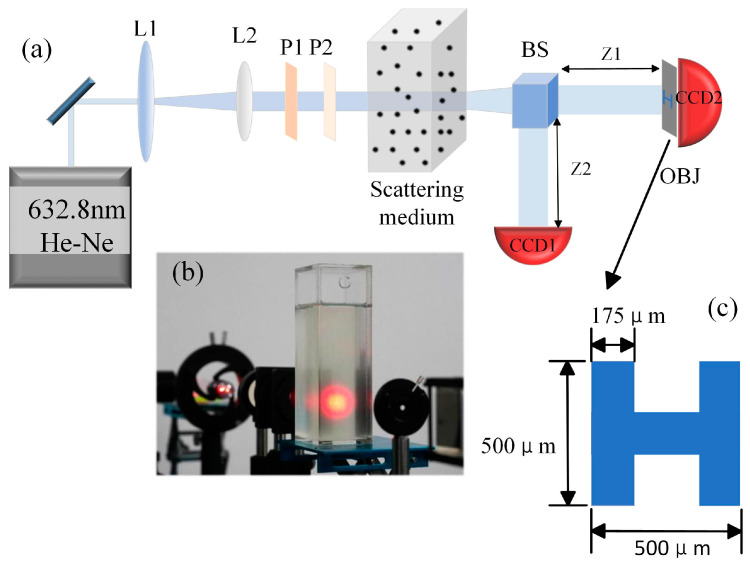
(**a**) Self-modulated experimental schematic diagram. (**b**) Scene of the laser beam passing through the scattering medium. (**c**) Pattern of the object.

As shown in Figure 2a, the experimental setup of the He-Ne laser (λ = 632.8 nm) sequentially passes through the beam expander (L1), collimation lens (L2), and two polarizers (P1, P2) before incident to the scattering medium. Polystyrene particles (approximately spherical with a diameter of 10 μm) suspended in an oleic acid solution are used to simulate the effect of scattering media. Because the density of the two materials is approximate (the density of oleic acid is 0.89 g/cm^3^ and the density of polystyrene particles is 1.05 g/cm^3^), the particles can be suspended in oleic acid, and the Brownian motion can last for a long time. The scattering intensity can be adjusted by changing the particle concentration.

After passing through the scattering medium, the laser beam is divided into two identical beams by a 50:50 beam splitter (BS). The light in the reference arm is recorded using the CCD1 (MERCURY2 Industrial Camera, Daheng optics, Beijing, China). For simplicity of alignment, another identical camera, CCD2, collects the transmitted light passing through the object in the object arm. At this time, CCD2 is used as a bucket detector [33,34]. The scene when the laser beam passes through the scattering medium in the experimental apparatus is shown in Figure 2b. The object (metal mask “H” in Figure 2c) is placed in the optical imaging path near CCD2, meanwhile ensuring that the distances from the BS to the two detectors are the same (x1 = x2). The sampling rate of the detector is set at 30 milliseconds per frame, and the image size for calculation is 256 × 256 pixels.

## 3. Results

### 3.1. Relationship between Random Speckle Pattern and Particle Concentration

According to the size of the scattered particles and wavelength of the light source, atmospheric scattering can be classified into Rayleigh scattering, Mie scattering, and non-selective scattering. Gong and Han proposed an improved correlation imaging method that could improve the imaging quality of Mie scattering media [25]. Bina et al. proposed using differential ghost imaging with the back scattering structure to reconstruct objects immersed in Rayleigh scattering turbid media [35].

A non-sequential bulk scatter model was established to simulate the scattering of the expanded laser beam as it passed through the solution containing particles [36,37]. The simulation parameters were set as follows: the wavelength of the laser beam was 632.8 nm, the number of analyzed rays was 10,000, the scattering area was a rectangular volume (20 × 20 × 10 mm), the refractive index of the filling material (oleic acid) in the rectangle volume was 1.4585, the refractive index of the particle (polystyrene microsphere) was 1.60, and the particle diameter was 10 microns. According to the ratio of particle radius to wavelength (2πr/λ ≈ 49.6), the simulation was based on Mie scattering [38,39,40]. A rectangle detector with pixel size 100 × 100 was placed on the scattering rectangular volume’s rear surface to observe the intensity distribution of transmitted light.

**Figure 3:**
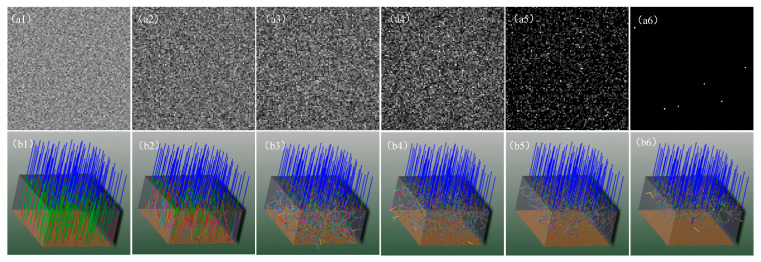
From (**1**) to (**6**), the strength of scattering is increasing. (**a1**–**a6**) Intensity distribution received by the detector. (**b1**–**b6**) Sequentially corresponding simulation models. The density of particles is (**a1**,**b1**) 5 × 10^4^ cm^−3^, (**a2**,**b2**) 5 × 10^5^ cm^−3^, (**a3**,**b3**) 5 × 10^6^ cm^−3^, (**a4**,**b4**) 1 × 10^7^ cm^−3^, (**a5**,**b5**) 1.5 × 10^7^ cm^−3^, and (**a6**,**b6**) 2 × 10^7^ cm^−3^.

**Figure 4:**
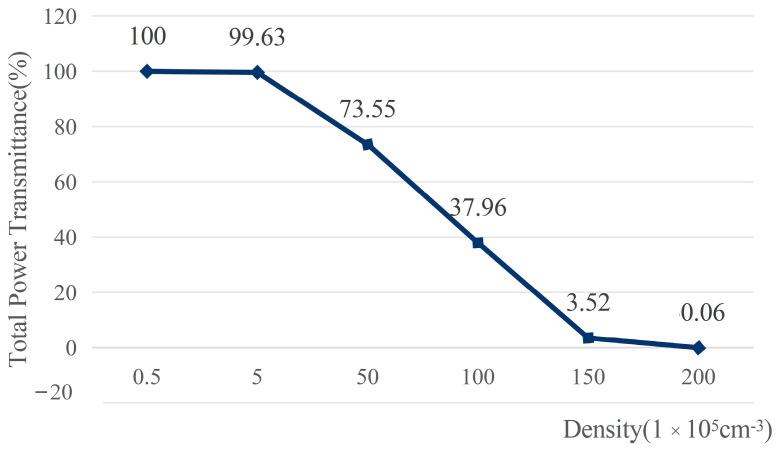
The total intensity transmittance corresponding to each concentration.

In order to investigate the relationship between random speckle pattern and particle concentration, experiments with different particle concentrations (the number of particles contained per unit volume) while other parameters were fixed were conducted. Figure 3a displays the intensity distribution of the laser beam that the detector received following ray tracing, Figure 3b presents the corresponding simulation models, and Figure 4 plots the relationship between the total intensity transmittance received by the detector and the particle concentration. It can be observed that the light transmittance gradually declines with a linear rise in particle concentration, and the detector’s total intensity is also reduced linearly. The simulation results show that the scattering effect is not apparent when the particle concentration is low. If the concentration of particles is too high, the scattering effect is so strong that most of the light cannot pass through the rectangular volume to reach the detector. Therefore, only when the laser beam passes through a scattering environment with a certain concentration range will it produce a random dynamic speckle suitable for ghost imaging.

### 3.2. Properties of SMGI Light Source

A thermal light source is a prerequisite for GI. In conventional GI experiments, the pseudo-thermal light is typically realized using a rotating ground glass. In order to investigate the property of the light modulated by the scattering medium, experiments and data analysis for light intensity distribution in time and space were conducted [41,42].

**Figure 5:**
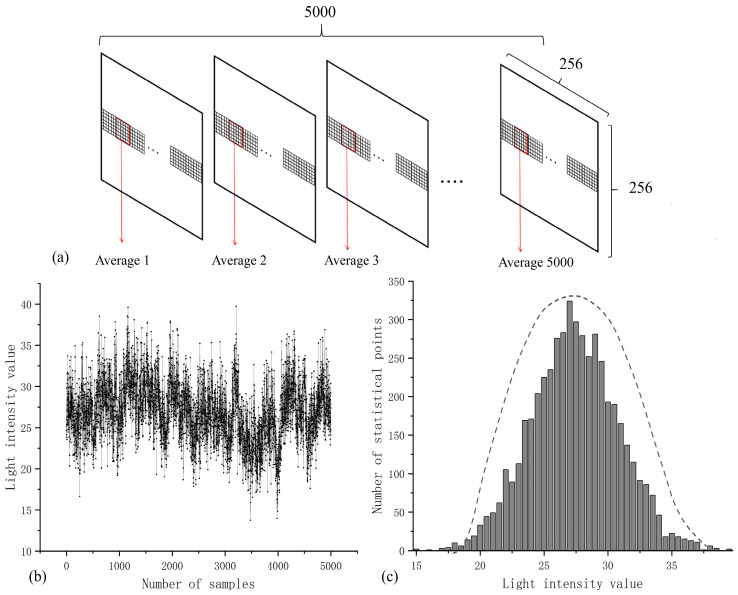
(**a**) The principle diagram of the statistics. (**b**) Statistical results of average light intensity. (**c**) The complex amplitude of the light field is approximately Gaussian random distribution in time.

In total, 5000 time-continuous reference light samples (recorded using CCD1) were taken to calculate the average light intensity value of 6 × 6 pixels in the same area, respectively. The principle is shown in Figure 5a,b, with data for 5000 average light intensity values, where the x-coordinate represents the number of successive samples and the y-coordinate is the corresponding average light intensity value. Figure 5c shows the quantity statistics of the light intensity value. The statistical results show that the complex amplitude of the light field generated by the self-modulation of the scattering medium is approximately Gaussian random distribution in time.

**Figure 6:**
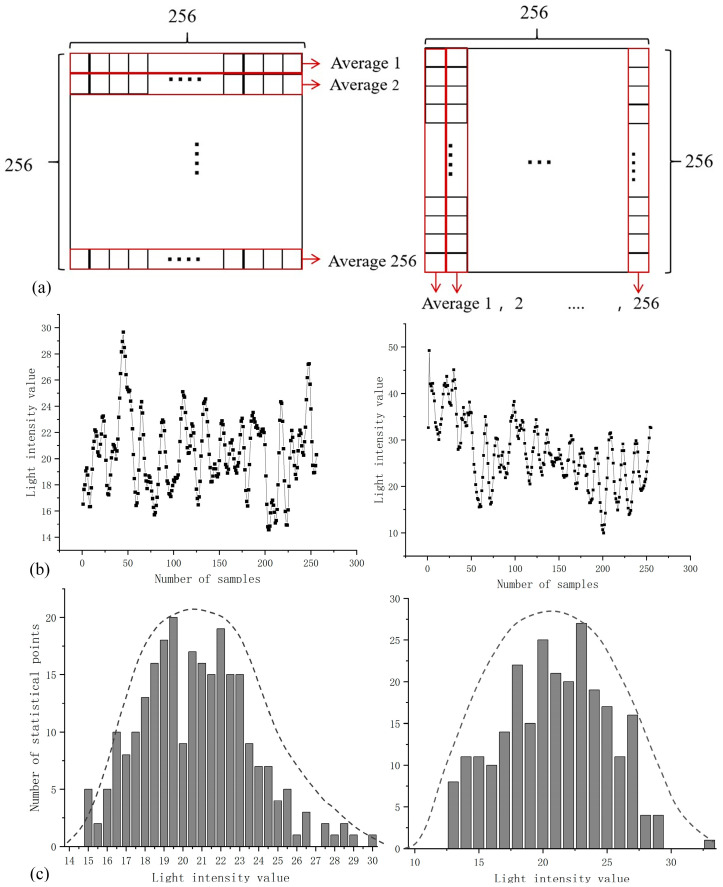
(**a**) The principle diagram of the statistics. (**b**) Statistical results of average light intensity. (**c**) The complex amplitude of the light field is approximately Gaussian random distribution in the transverse and longitudinal space.

A reference light (recorded using CCD1) was randomly selected, and the light intensity values of 256 pixels in each row and column in the sample were averaged. The principle is shown in Figure 6a,b, with the statistical results of the average light intensity values corresponding to the above 256 rows and columns, respectively. Figure 6c shows the quantitative statistics of the light intensity value. The statistical results show that the mean complex amplitudes of the light field generated by the self-modulation of the scattering medium in the transverse and longitudinal space are approximately Gaussian random distribution.

The statistical results show that the light source generated through self-modulation of the scattering medium has strong fluctuation in both time and space, and the complex amplitudes of both light fields obey Gaussian random distribution, which is the same as the thermal light source.

### 3.3. SMGI Performance

**Figure 7:**
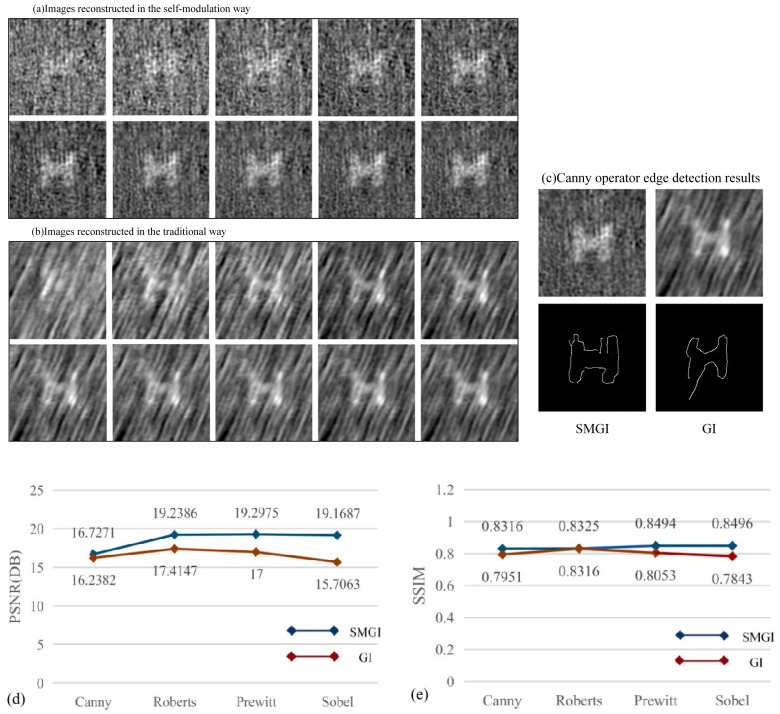
(**a**,**b**) Reconstructed images of SMGI and traditional GI, respectively. The results were reconstructed with 1000, 2000, 3000, 4000, 5000, 6000, 7000, 8000, 9000, and 10,000 frame samples in turn. (**c**) Canny edge detector result. The PSNR (**d**) and SSIM (**e**) line charts of SMGI and GI.

In Figure 7, the reconstructed images with a self-modulated pseudo-thermal source and a ground-glass-modulated pseudo-thermal source are presented individually. As a comparison between Figure 7a,b, it is observed that the image reconstructed using traditional ghost imaging has apparent “fluidity”, and the image reconstructed through self-modulation is less noisy. This verifies that the pseudo-thermal light generated by the scattering medium fluctuates more evenly in time and space than that generated by ground glass.

In order to further compare the performance of SMGI and GI, the edge characteristics of the “H” figure reconstructed using the two methods were quantitatively analyzed. Peak signal to noise ratio (PSNR) and structural index measurement (SSIM) were used as evaluation indexes. The PSNR is most commonly used to measure the reconstruction quality in image compression. SSIM is a universal objective image quality index that is easy to calculate and applicable to various image processing applications [43]. Firstly, two reconstructed images with 10,000 frame samples were average filtered [44], and edge detection was carried out using Canny, Roberts, Prewitt, and Sobel edge detectors [45]. The parameter values of each edge detector were adjusted several times to retain the extracted target graph information to the maximum extent. The parameters of the four edge detectors were determined to be 0.6, 0.014, 0.02, and 0.019, respectively. The PSNR and SSIM of the two groups of edge images were calculated. Figure 7c shows the results obtained using the Canny edge detector, Figure 7d shows the relationship between the two groups of image PSNRs, and Figure 7e shows the relationship between the two groups of SSIMs. The experimental results demonstrate that the performance of SMGI is better than that of GI.

**Figure 8:**
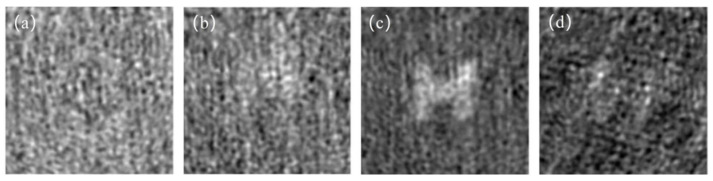
Concentration of particles ((**a**) 0.05 mg/cm^3^, (**b**) 1.0 mg/cm^3^, (**c**) 1.5mg/cm^3^, (**d**) 2.0mg/cm^3^).

As shown in Figure 8, the influence of particle concentration on SMGI was investigated. First of all, the reconstructed images with 10,000 frame samples for four different particle concentrations are presented. It is observed that the image quality is poor when the particle concentration is low. With the increase in particle concentration, the image gradually becomes clearer. However, when the concentration is too high, the image quality decreases again, which is very consistent with the simulation results above.

Based on the simulation and experimental results, when particles with a concentration ranging from 1.3 to 1.7 mg/cm^3^ and a travel length of 10cm are in a scattering medium (the diameter of the particles is 10 μm), a clear image can be reconstructed with 10,000 frame pictures. The concentration range will vary when the particle diameter and refractive index change. If the particle concentration is too low, the scattering effect is negligible. It cannot generate a randomly distributed illuminating light beam. Meanwhile, if the concentration is too high, the scattering effect becomes excessively strong, and the transmittance becomes too low; there is too little light able to reach the object.

## 4. Discussion

When there is a scattering medium in the optical transmission path, traditional imaging cannot establish the corresponding relationship of the light field. Ghost imaging belongs to second-order intensity correlation imaging, which has been proven to have strong anti-interference ability, a simple optical path, low cost, and other characteristics. It provides a new idea for obtaining clear object images in scattering media and has been widely used and become a hot research topic in related fields.

This paper demonstrated improved ghost imaging, which we called self-modulated ghost imaging (SMGI). SMGI can take advantage of the dynamic scattering medium that originally affects the imaging quality and generate pseudo-thermal light through the dynamic scattering of free particles’ Brownian motion in the scattering environment for imaging. The main contribution of this work includes the following three points. Firstly, as shown in Figure 3 and Figure 4, theoretical analysis and simulation established the relationship between the random speckle pattern and particle concentration of SMGI, which verified that self-modulation within a certain particle concentration range could produce random dynamic speckle suitable for ghost imaging. When light travels in a scattering medium with a travel length of 10 cm and particle concentration ranging from 1.3 to 1.7 mg/cm^3^, a clear image can be reconstructed with 10,000 frame pictures. The experimental results, as shown in Figure 7, are very consistent with the simulation results above. Secondly, from the statistical results in Figure 5, we can find that the properties of self-modulating pseudo-thermal light are similar to those of traditional GI, which can be used as an experimental light source for ghost imaging. Finally, Figure 1 and Figure 2 are schematic diagrams of the experimental device, and traditional GI and SMGI imaging experiments were carried out, respectively. The experimental results are shown in Figure 6 and show that SMGI can reduce the fringe interference caused by the rotating ground glass in traditional GI imaging. According to the evaluation indexes PSNR and SSIM, SMGI can improve the quality of reconstructed images.

Although there are some limitations in the experimental process and results of the improved GI proposed in this paper, SMGI has potential application prospects for the reconstruction of images in random dynamic high-scattering environments such as dense fog and turbid water, and SMGI provides a simple and effective method to generate pseudo-thermal light for ghost imaging in dynamic scattering media, which provides a new idea for the application of traditional GI in scattering environments.

## Data Availability

Data underlying the results presented in this paper are not publicly available at this time but may be obtained from the authors upon reasonable request.
